# Genome-wide associations for milk production and somatic cell score in Holstein-Friesian cattle in Ireland

**DOI:** 10.1186/1471-2156-13-21

**Published:** 2012-03-26

**Authors:** Brian K Meredith, Francis J Kearney, Emma K Finlay, Daniel G Bradley, Alan G Fahey, Donagh P Berry, David J Lynn

**Affiliations:** 1 Animal and Bioscience Research Department, Teagasc, Animal & Grassland Research and Innovation Centre, Grange, Dunsany, Co. Meath, Ireland; 2 Animal and Bioscience Research Department, Teagasc, Animal & Grassland Research and Innovation Centre, Moorepark, Co. Cork, Fermoy, Ireland; 3 Irish Cattle Breeding Federation, Bandon, Co. Cork, Ireland; 4 Smurfit Institute of Genetics, Trinity College Dublin, Dublin 2, Ireland; 5 School of Agriculture and Food Science, University College Dublin, Dublin 4, Belfield, Ireland

## Abstract

**Background:**

Contemporary dairy breeding goals have broadened to include, along with milk production traits, a number of non-production-related traits in an effort to improve the overall functionality of the dairy cow. Increased indirect selection for resistance to mastitis, one of the most important production-related diseases in the dairy sector, via selection for reduced somatic cell count has been part of these broadened goals. A number of genome-wide association studies have identified genetic variants associated with milk production traits and mastitis resistance, however the majority of these studies have been based on animals which were predominantly kept in confinement and fed a concentrate-based diet (i.e. high-input production systems). This genome-wide association study aims to detect associations using genotypic and phenotypic data from Irish Holstein-Friesian cattle fed predominantly grazed grass in a pasture-based production system (low-input).

**Results:**

Significant associations were detected for milk yield, fat yield, protein yield, fat percentage, protein percentage and somatic cell score using separate single-locus, frequentist and multi-locus, Bayesian approaches. These associations were detected using two separate populations of Holstein-Friesian sires and cows. In total, 1,529 and 37 associations were detected in the sires using a single SNP regression and a Bayesian method, respectively. There were 103 associations in common between the sires and cows across all the traits. As well as detecting associations within known QTL regions, a number of novel associations were detected; the most notable of these was a region of chromosome 13 associated with milk yield in the population of Holstein-Friesian sires.

**Conclusions:**

A total of 276 of novel SNPs were detected in the sires using a single SNP regression approach. Although obvious candidate genes may not be initially forthcoming, this study provides a preliminary framework upon which to identify the causal mechanisms underlying the various milk production traits and somatic cell score. Consequently this will deepen our understanding of how these traits are expressed.

## Background

Dairy production is an economically important sector of global agriculture with the top 10 leading dairy companies turning over in excess of $114 billion in 2009 [1]. Dairy cows account for 84% of global dairy output [1] so consequently there is great interest placed upon the production potential and health of these animals. Until recently, the majority of international dairy breeding programmes selected solely for increased milk production, however, breeding goals have diversified to include health and functional traits in an effort to minimise and reverse the decline in these traits [2]. Prominent among these health-related traits is mastitis (commonly measured using somatic cell score (SCS) as an indicator trait), which is one of the most important and costly production diseases in the dairy industry. Selection for improved milk production traits and reduced SCS (indicating increased mastitis resistance) can potentially be improved through the identification of quantitative trait loci (QTL) associated with these traits of interest by allowing geneticists to infer and comprehend the genetic and molecular mechanisms underlying the traits.

QTL associated with milk production and SCS have been extensively reviewed by, amongst others, Khatkar et al. [3] and Smaragdov et al. [4]. Milk production QTL have been reported most often on chromosomes 1, 3, 6, 10, 14 and 20 while SCS QTL were most frequently observed on chromosomes 5, 8, 11, 18 and 23. Many of the reviewed studies utilised family-based studies and microsatellite markers to identify areas of the genome associated with a particular trait, however, these studies were often limited by the relatively low number of genetic markers sparsely distributed across the genome.

The sequencing of the bovine genome and the subsequent HapMap project made large amounts of genetic markers available in the form of single nucleotide polymorphisms (SNPs). This massive increase in marker numbers allied with the emergence of high-throughout genotyping technologies allowed routine genome-wide association studies (GWAS) to be performed in cattle populations. GWAS allow screening of the genome utilising a large number of genetic markers spread across the entire genome to detect genetic variants associated with a particular disease or trait. The majority of recent GWAS employ SNPs as genetic markers. SNPs may not themselves be responsible for the variation observed in a trait, however, due to their close proximity to un-genotyped causal variants they have been co-inherited and so can act as proxies for the unknown causal variants [5]. In this way, SNPs significantly associated with a disease or trait may indicate a region of the genome which harbours genetic variants influencing the expression of that disease or trait. In general, GWAS studies act as an initial screening tool, from which significantly associated regions can be further refined using a higher marker density (potentially by re-sequencing), with the ultimate goal of identifying candidate genes believed to underlie the trait(s) of interest. Candidate genes can then be characterised further in an attempt to identify the functional mechanisms underlying a trait. This will lead to a greater understanding of molecular basis and regulation of the trait in question. A number of recent studies in dairy cattle have detected associations with production and functional traits using a GWAS approach [6-8].

In general, GWAS studies to date have identified important genomic regions using single locus association methods whereby each individual SNP is tested for association with the trait of interest. This method has proved useful in its straight-forward implementation and interpretation, however, it has been hampered by the large number of false positive results produced due to the large number of markers and hence individual statistical tests. This problem has been somewhat addressed through multiple correction techniques such as the false discovery rate (FDR) [9], yet markers with small effects are liable to be lost in this manner. Multi-locus and validation-based approaches stand as possible alternatives to tackle the amount of false positives produced from a GWAS. An example of a validation-based approach is a two-stage GWAS where an initial genome-wide scan is performed in a large group of animals to identify a subset of significant SNP. This is then replicated in an independent population of animals to validate the significant associations. Such an approach has been used in a GWAS investigating bull fertility [10].

Irrespective of the statistical approach or study design used, most QTL studies to-date in cattle have largely been undertaken using phenotypic data originating from high-input, concentrate-based dairy production systems. Genotype by environment (GxE) interactions have, however, been reported between high- (i.e. animals predominantly kept in confinement and fed a concentrate-based diet) and low-input production (i.e. animals fed a predominantly forage-based diet in a pasture-based production system) systems [11-14]. In general, interaction effects tend to be scaling effects where animals retain the same ranking across different environments, however, re-ranking of bulls between pasture and total mixed ration feeding systems has been reported [15]. Indeed, Interbull [16] issues separate sets of results of it's Multiple Across Country Evaluation to each participating country due to potential re-ranking of sires via GxE interactions. Consequently, we hypothesised that some QTL for milk production traits and/or SCS may vary across may vary across different production systems.

The objective of this study was to identify regions of the genome associated with milk production traits and SCS in cattle fed a basal diet of grazed grass (low-input system) using single SNP regression in a dataset of 914 Holstein-Friesian sires. A multi-locus, Bayesian approach in the same population and a single SNP regression analysis in a separate population of Holstein-Friesian cows were conducted to provide further support to the associations.

## Results

### Significant associations

**Figure 1:**
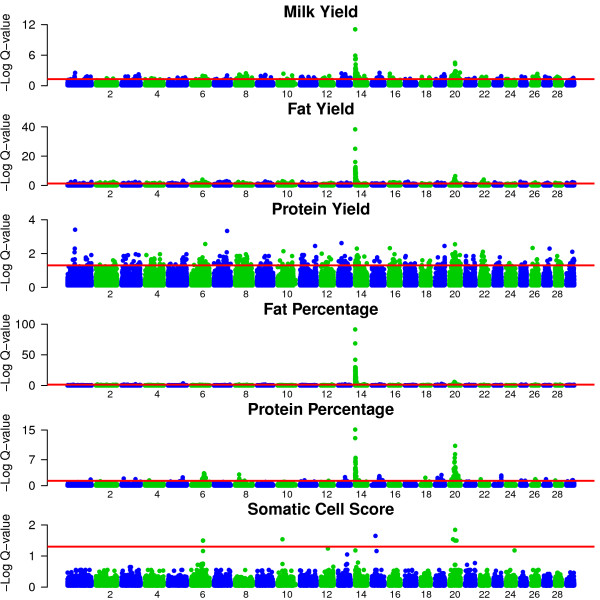
**Location of significant SNPs from the single SNP regression in the sires for all traits**. Associations (-log Q-value) of all SNPs using the single SNP regression model in the sires for each trait across all 29 autosomes. The minus log of the q-value (y-axis) is plotted for each chromosome (Chr) (x-axis). The 5% significance threshold is indicated with a red line.

Two populations of 914 Holstein-Friesian AI sires and 493 Holstein-Friesian cows were used to quantify associations between genotypic and phenotypic data. A summary of the various phenotypes and the correlations between them in the sire dataset are detailed in Table 1 and Table 2 respectively. The corresponding information for the cows is detailed in Additional file 1 and Additional file 2 respectively. A set of haplotype blocks was defined using the 40,668 SNPs from the sires. Using these SNPs, 10,958 haplotype blocks were defined which accounted for ~1 Gb of the genome. On average, the blocks were 97,006 Kb in length and contained 2.84 SNPs; the majority of SNPs (n = 31,157 SNPs) were located within haplotype blocks. The remaining 9,511 SNPs were not located within any haplotype block. Two statistical approaches, a frequentist single SNP regression approach and a multi-locus Bayesian method, were used to detect significant associations with milk, fat and protein yield, milk fat and protein percentage and somatic cell score (SCS). In total, 1,529 and 37 associations were detected in the sires using a single SNP regression and a Bayesian method respectively. Of these, 276 associations were found to be novel (i.e. had a q-value ≤ 0.05 and were located outside any known QTL regions for the particular trait) in the sires using the single SNP regression approach. There were 103 associations in common between the sires and cows across all the traits. The results of the Bayesian analysis were tested for robustness to the prior probability of a SNP being associated with the phenotype. Only SNPs with a posterior probability of association ≥ 0.8 for two out of the three priors were considered significant. The results using different priors were generally consistent within a trait with the same SNP or a SNP in the same region of the genome indicated as significant across all priors. This Bayesian approach allowed a multi-locus statistical approach while also providing the opportunity to validate any significant associations found using the single SNP regression approach in the same population of sires (figure 1). Indeed numerous SNPs were detected as significant using the Bayesian approach which were also significant using the single SNP regression.

**Table 1:** Summary statistics for the phenotypic data in the sires

	Trait	N	Mean	σ
	Milk Yield (kg)	914	158.3	231.2

	Fat Yield (kg)	914	6.1	7.6

Sires	Protein Yield (kg)	914	5.8	6.6

	Fat Percentage (kg) (× 1000)	914	4.9	142.3

	Protein Percentage (kg) (× 1000)	914	-0.96	82.7

	Somatic Cell Score (log_e _SCC) (× 1000)	773	34.2	104.9

Summary statistics include the total number of phenotypic records (N), mean and standard deviation (σ) for each trait in the sires. Phenotypes in the sires are expressed as daughter yield deviations on a PTA scale

**Table 2:** Pearson correlations between phenotypes for the sires

Trait	Milk Yield	Fat Yield	Protein Yield	Fat %	Protein %	SCS
Milk Yield		0.57	0.87	-0.60	-0.70	0.12

Fat Yield	0.57		0.75	0.34	-0.02	0.16

Protein Yield	0.87	0.75		-0.25	-0.23	0.17

Fat %	-0.60	0.34	-0.25		0.76	0.01

Protein %	-0.70	-0.02	-0.23	0.76		0.01

SCS	0.12	0.16	0.17	0.01	0.01	

Fat % = fat percentage; Protein % = protein percentage; SCS = somatic cell score

Correlations between phenotypes in the sires expressed as daughter yield deviations on a PTA scale

The use of a single SNP regression approach in a population of 493 Holstein-Friesian cows endeavoured to replicate any previous associations in a semi-independent population. The average additive relationship among the sires was 0.046, was 0.040 among the cows, and was 0.038 between the sires and cows. Significant associations detected for the two statistical approaches in the sires and the single SNP regression in the cows are detailed for each trait in Additional file 3.

A number of SNPs were significantly associated with more than one trait. We combined all significant associations (i.e. from both sire statistical models and the cow validation dataset) detected for a trait and identified 232, 83, 52 and 3 SNPs which were significantly associated with 2, 3, 4 and 5 traits, respectively (Additional file 3).

### Milk yield

Using the single SNP regression approach, 370 SNPs were identified as being significantly associated (q ≤ 0.05) with milk yield at the 5% FDR in the sire population. The majority of these SNPs (311/370) were located in known QTL regions for milk yield [17-20]. Several genomic regions contain clusters of SNPs associated with the trait, including several on chromosome 14 in close proximity to the DGAT1 gene, which is known to affect milk yield [21-23]. Indeed, the top-ranked SNP for milk yield (q = 8.33 × 10^-12^) was located 146 base pairs from the DGAT1. A cluster of SNPs on chromosome 20, in a region also known to be associated with milk yield [18,19,24], was also identified. Notably, 59 SNPs, which do not appear to be located within known QTL regions for milk yield, were detected on nine different chromosomes (Additional file 3). However, none of these novel SNPs were significant using the Bayesian model or in the cows. Of potential interest was a cluster of significant SNPs on chromosome 13 from ~45-49 Mb which contained numerous SNPs of moderate association (lowest q-value = 5.14 × 10^-6^). The genes in this region are listed in Additional file 3.

There were two SNPs significantly associated with milk yield using the Bayesian approach in the sires (Additional file 3). These two SNPs, rs42211964 located ~27 Mb on chromosome 8 and rs109421300 located ~0.4 Mb on chromosome 14 were also significantly associated with milk yield using the single SNP regression in the sires. Both of these occurred in known QTL regions [17,19] with the latter in close proximity to the DGAT1 gene known to affect milk yield in dairy cattle [21].

### Fat yield

A total of 370 SNPs were significantly associated (q ≤ 0.05) with fat yield using the single SNP regression in the sires (Additional file 3). As with milk yield, the majority (281/370) of these were located in known QTL regions for fat yield [22,25-27]. Additionally, the most significantly associated SNPs were also located close to the DGAT1 gene [21] with one SNP in this region, rs109421300 (q = 5.15 × 10^-39^), being the most significant association for fat yield. However, 89 significant SNPs were detected outside known QTL regions from Cattle QTLdb [28] and were considered novel (i.e. a q-value ≤ 0.05 and located outside any known QTL regions for fat yield). The most interesting and strongly-associated of these novel SNPs were two clusters of SNPs at 34 Mb and 39 Mb which are in close proximity to the GHR and PRLR genes, respectively. Of the 89 novel SNPs, one SNP in a similar region of chromosome 20 was also significant in the Bayesian method. We were unable to replicate these associations in the cow dataset, possibly due to the reduced power in that data (see discussion).

Using the Bayesian approach, 12 SNPs across eight chromosomes were significantly associated with fat yield in the population of 914 sires. Several of these SNPs (4/12), located on chromosomes 5, 7, and 14, were assigned posterior probabilities of association ≥ 0.8 using all three Bayesian priors and were located within known QTL regions [22,29,30]. One of the eight SNPs located in known QTL regions, rs29016908 on chromosome 5, was positioned 28 kb from the EPS8 gene which binds with the EGFR gene to alter responsiveness to EGF [31]. EGF is believed to affect various milk production traits [32]. An additional four significant SNPs, located on chromosomes 11, 20 and 25, did not overlap with known QTL regions and appear to be novel. Over half (7/12) of the SNPs significantly associated with fat yield in the Bayesian approach were also significant in the single SNP regression in the sires, providing further evidence of their association.

### Protein yield

Using the single SNP regression model, 385 SNPs were significantly associated (q ≤ 0.05) with protein yield in the sires. Most of these SNPs (305/385) were found to lie within known QTL regions for protein yield [26,33,34]. Unlike milk and fat yield, the largest associations were not focused on chromosomes 14 and 20 but were instead distributed across numerous other chromosomes. The most significantly associated SNP across all chromosomes was rs42327956 (q = 3.86 × 10^-4^) located at ~50,6 Mb on chromosome 1. Several significantly associated SNPs were clustered on chromosome 1 in a region which overlaps with known QTL regions for protein yield [33-35]. The genes in this region are supplied in Additional file 3. There were 80 significant SNPs located outside known QTL regions with a cluster of SNPs located on chromosome 8. However none of these 80 novel SNPs were significant in the Bayesian approach or in the cows.

There were two SNPs significantly associated with protein yield in the sire population using the Bayesian approach. These SNPS, located on chromosomes 11 and 27 were both located in known QTL regions for protein yield [34,36]. One SNP, rs41257411 located on chromosome 27, was also significantly associated with protein percentage using the single SNP regression in the population of 914 sires.

### Fat percentage

Using the single SNP regression approach in the sires, a total of 216 SNPs were significantly associated (q ≤ 0.05) with fat percentage with 199 of these located within known QTL regions for fat percentage [22,24,35,37,38]. The location of significant associations was similar to that of fat yield with strongest associations harboured in a region encompassing the first 6 Mb of chromosome 14. Similar to milk yield, the strongest association (q = 3.91 × 10^-92^) was detected for a SNP close to the DGAT1 gene, a gene known to heavily affect milk fat percentage [21,23]. Several significantly associated SNPs were also detected in a segment of chromosome 20 from ~34-37 Mb which are located close to the GHR gene, which is also known to affect milk production traits [38]. There were 17 potentially novel significant SNPs (q ≤ 0.05) detected outside known QTL regions for fat percentage. Of particular interest was a number of novel SNPs on chromosome 13 located at 46 Mb. A potentially novel association for milk yield was also detected in this region. However, none of these novel SNPs were detected as significant using the Bayesian approach or in the cows.

A total of 12 SNPs were significantly associated with fat percentage using the Bayesian approach in the sire population. Nine of these SNPs were located in known QTL regions [24,35,39]. Two SNPs, rs109421300 on chromosome 14 and rs110482506 on chromosome 20 located in close proximity to the DGAT1 and GHR genes, respectively. Surprisingly, only two SNPs significantly associated with fat percentage were also significantly associated with fat yield using the Bayesian approach, however, several significant SNPs were located in similar genomic regions to those associated with fat yield. Furthermore, three SNPs located on chromosomes 9, 21 and 27 did not occur in any known QTL regions for fat percentage. Of the 12 significant SNPs detected in the Bayesian approach, four of these were also significant for fat percentage in the sires using the single SNP regression approach.

### Protein percentage

Using the single SNP regression in the sires, there were 229 SNPs significantly associated (q ≤ 0.05) with protein percentage of which 204 SNPs were within known QTL regions for protein percentage [19,24,26,30]. Like the majority of the milk production traits, clusters of associations were located on chromosomes 14 and 20. A cluster of significant SNPs in a region 0-6 Mb on chromosome 14 contained the strongest association (q = 7.44 × 10^-16^) for a SNP close to the DGAT1 gene which has been shown to affect protein percentage [22,23]. In addition, a segment of chromosome 20 from 29 to 40 Mb, which contains the GHR gene, harboured a large number of significantly associated SNPs. Several strong associations were also detected on chromosome 6 between 80 and 90 Mb, a region that contains the Casein gene cluster which has been associated with changes in the protein composition of bovine milk [40]. However, all the aforementioned associations were located within known QTL regions for protein percentage [19,24,26,41,42]. An additional 25 significant SNPs were found outside known QTL regions and these occurred across 10 different chromosomes. Of these rs41573791 (q = 9.94 × 10^-4^) on chromosome 8 was the most strongly associated with protein percentage. In addition a number of novel SNPs located at 50 Mb on chromosome 15 were also moderately (q-value = 0.002) associated. One of these 25 novel SNPs, located on chromosome 5, was also detected as significant when using the Bayesian approach, however, none were significant in the cows.

There were eight SNPs significantly associated with protein percentage using the Bayesian approach in the sires with five of these SNPs overlapping known QTL regions [19,26,43]. The remaining three SNPs, located outside known QTL regions for protein percentage, were located on chromosomes 5, 12 and 18. Furthermore, five out of the eight significantly associated SNPs were also significantly associated with protein percentage using the single SNP regression in the sire population.

### Somatic cell score

Only nine SNPs were significantly associated (q ≤ 0.05) with SCS using the single SNP regression in the sires, three of these were located within known QTL regions for SCS. These three SNPs were located on chromosomes 6 and 10 [34,44,45]. The remaining six SNPs, located outside known QTL regions for SCS, were spread across chromosomes 6, 15 and 20 with the most significant (q = 0.014) of these located on chromosome 20. None of these six novel SNPs were significant in the Bayesian approach or in the cows. All SNPs significantly associated with SCS are listed in Additional file 3 along with genes close to or overlapping them.

Only a single SNP, rs41590209 located at ~97 Mb on chromosome 4, was significantly associated with SCS using the Bayesian approach and this fell into a known QTL region for SCS [46]. However, neither this SNP nor any SNP on chromosome 4 was significantly associated with SCS using the single SNP regression in the population of 773 sires.

## Discussion

The objective of this study was to identify QTL associated with milk production traits and SCS in Holstein-Friesian cattle from a low-input production system. Both a frequentist and a Bayesian statistical approach were employed to test for association between genotypes and phenotypes. The QTL identified using both the sire and cow populations were spread across all 29 autosomes; the location and frequency of these QTL were in general agreement with those previously reported [3,4].

A large number (1,529) of significant associations were detected across all traits. The majority of these significant associations were located within known QTL for the trait of interest. This shows that our methodology is effective in detecting associated regions of the genome. Also, our findings will help to further refine QTL regions previously detected with microsatellites [47]. The detection of a large number of known QTL regions in our study would suggest that a large number of QTL that are important in high-input, confinement, concentrate-based systems are also important in low-input, pasture-based systems such as ours. In spite of this, 276 novel SNPs were detected in the sires using the single SNP regression approach. Of these novel SNPs, a number of promising clusters of SNPs were identified for each trait which may indicate potential new QTL regions. These regions include an area of chromosome 13 significantly associated with milk yield and fat percentage. Also, significant novel associations were detected on chromosome 20 for fat yield and somatic cell score close to the GHR and PRLR genes reported to be associated with milk production traits and SCS [38,48]. In addition, particular areas of interest were separately detected for protein yield and percentage on chromosomes 8 and 15, respectively. These genomic regions may consist of QTLs that are unique to or advantageous in a low-input system such as ours.

Several significant associations, both within and outside known QTL regions, were detected for SCS. However, associations were considerably less numerous and weaker compared with those for the milk production traits. This may have been due to several inherent problems with the SCS phenotype resulting in reduced power to detect associations. Firstly, the reliability of the SCS proofs, which is an indicator of the amount of information available for an animal, was lower than that of the milk production traits in both the sires and cows. Decreased reliability of SCS means greater uncertainty as to the true breeding value of the animal for that trait. Furthermore, in the sire population, there were 138 fewer animals used to test for associations with SCS which would also decrease the power to detect significant associations. In addition, the lower heritability of SCS when compared to that of the milk production traits may also contribute to the weaker associations identified for SCS (i.e. a greater number of animals may be required for SCS

The Bayesian analysis used provided a number of advantages/alternatives to the standard single SNP regression approach. This Bayesian approach fits all markers in the analysis simultaneously and it was noticeable that this approach detected only 1-2 significantly-associated SNPs where the single SNP regression detected a cluster of numerous significantly-associated SNPs (i.e. on chromosome 14 for fat percentage). Additionally, the ability to allow *a priori *information to be factored into the statistical model appears to have merit where different traits mat be controlled by varying numbers of genetic variants [49]. The prior appears to be robust, with similar genomic regions detected as significant even when using different priors.

Genome-wide association studies are susceptible to detection of false positives due to the large number of statistical approaches being performed. One method to confirm or validate a SNP association/QTL is via replication of the association in a separate population as the probability of detecting the same associated variant in two separate populations is small [50]. Our use of a separate population of Holstein-Friesian cows allowed validation of a number of associations from the sires, however, the size of this population was probably insufficient to validate SNPs of smaller effect (i.e. power was lower). Also the reliabilities of all traits in the cows were much lower than those of the sires resulting in potentially less accurate phenotypes to quantify the associations.

A number of SNPs in this analysis were significantly associated with more than one trait suggesting that genes with pleiotropic action may have been detected. Typically, in this study, a SNP affected multiple production traits with no association with SCS. Examples of this are three SNPs which were significantly associated with all five production traits (Additional file 3). This indicates that certain regions of the genome may affect various different production-related traits and this should be taken into consideration when selecting animals for a particular breeding goal. In addition, four SNPs were significantly associated with a production trait and SCS, in particular three SNPs on chromosome 20 were associated with a concurrent decrease in milk yield and SCS. This observation agrees with the well-known positive correlation that exists between milk yield and SCS [51]. Of these three SNPs, two lie in close proximity to the PRLR gene which has been reported to be associated with milk production [48] and changes in SCC [52]. QTL regions such as this may help elucidate how to select for increased milk yield without the associated detrimental effect on resistance to mastitis.

## Conclusion

A large number of significant associations were detected in this analysis which either overlapped known QTL regions or were novel associations found outside these QTL regions. Those associations found within known QTL regions can help to further refine large QTL regions potentially leading to the discovery of the underlying causal mechanisms. A number of strongly-associated regions were detected among the 276 novel SNPs found outside known QTL regions. These genomic regions may indeed be unique to a low-input, pasture-based system. These QTL regions can form the basis towards identifying potential candidate genes and genetic variants underlying the traits of interest.

## Methods

### Sire DNA extraction

Thawed frozen semen was washed twice in phosphate-buffered saline (pH 7.4), and cell pellets were harvested via centrifugation and re-suspended in 450 μL of pre-warmed extraction buffer (10 mM Tris, pH 8.0; 10 mM EDTA, pH 8.0; 1% SDS; 100 mM NaCl); 15 μL of 2-mercaptoethanol was added. Samples were incubated at 55°C for 15 minutes followed by the addition of 10 μL of proteinase K (20 mg/mL). Lysis occurred following overnight incubation at 60°C. DNA was then extracted using the Maxwell instrument (Promega Corp., Madison, WI) according to the manufacturer's instructions. Details of the DNA extraction method used in the cow dataset are available in Additional file 4.

### Sire genotypic and phenotypic data

In total 54,001 biallelic SNPs for 1,957 Holstein-Friesian AI sires with progeny in Ireland were genotyped in this study; the sires were representative of the germplasm used in Irish dairy herds in past years. All animal procedures were carried out according to the provisions of the Irish Cruelty to Animals Act (licenses issued by the Department of Health and Children). Sires were genotyped using the Illumina BovineSNP50 Genotyping Beadchip (Illumina Inc., San Diego, CA). SNP positions were based on BTAU 4.0. The 2,419 SNPs that were on the X chromosome or whose positions on the genome were unknown were eliminated from the dataset. SNPs on the X chromosome were removed due to large-scale discordance between SNP genotypes and animal sex (i.e. numerous SNP on the X chromosome, outside the pseudoautosomal regions, were called as heterozygous in male animals) indicating poor SNP calling. A further 230 SNPs were discarded that did not conform to Mendelian inheritance patterns between sire and son based on analysis of a larger dataset of animals [53]. The remaining 51,352 SNPs were subjected to additional SNP editing in the following order; SNPs were removed if they were monomorphic (n = 4,772), had a minor allele frequency ≤ 5% (n = 5,087), if greater than 5% of SNP calls were missing (n = 770), if there was poor SNP clustering (n = 14) or the proportion of heterozygotes for a SNP was > 90% (n = 41). Following all edits 40,668 SNPs remained.

Daughter yield deviations (DYD) for milk yield, fat yield, protein yield, fat percentage, protein percentage and SCS along with their respective reliabilities were available from national genetic evaluations undertaken in January 2010 by the Irish Cattle Breeding Federation (ICBF). Summary statistics for the phenotypic data is presented in Table 1. DYD for the aforementioned milk production traits and average SCS (i.e., log_e _SCC) are estimated in Ireland using a repeatability animal model across the first five lactations [54]. The parental contribution to the reliability of each trait was removed using the method described by Harris and Johnson [55]. Only sires with an adjusted reliability of ≥ 80% for milk production or ≥ 70% for SCS were retained. In total 914 sires met these criteria for inclusion in the analysis of milk production and 773 sires were included for SCS. Details regarding the genotypic and phenotypic data in the cows can be seen in Additional file 4.

### Statistical analyses

A single SNP regression model and a Bayesian method were both used to detect regions of the genome associated with milk production traits and SCS. In both approaches the dependent variable was either the DYD (sires) or YD (cows). SNPs were included in all models as continuous variables. A pedigree file, containing at least the previous four generations of the genotyped animals ancestors, was generated for each of the sire and cow datasets, respectively. The pedigree consisted of 5,479 animals for the sires and 4,476 individuals in the cows. The relatedness within and between the sires and cows was determined by calculating the average additive relationship between animals using the available pedigree information.

### Single SNP regression model

The dependent variable (i.e. the respective trait (daughter) yield deviation) was regressed on each SNP individually in a mixed animal model accounting for relationships among animals using the additive genetic relationship matrix in ASReml [56]. The individual animal was included as a random effect. Estimates of SNP effect and the associated standard error were recorded for each SNP. A false discovery rate (FDR) approach as described by Storey and Tibshirani [9], was used to correct for multiple testing. This was done in R version 2.12.0 [57] using the q-value package [58] to calculate q values (q). Q-values ≤ 0.05 were defined as significant.

### Bayesian model

The Bayesian method used was BayesB as described by Meuwissen et al. [59] modified to allow weightings based on the reliability of the dependent variable. An inverse chi-square distribution (v = 4.234 S = 0.0429) was used as a prior to reflect the assumption that, within a given population, only a subset of all loci truly affect the phenotype while the remainder have no effect at all on the phenotype. A prior value was assigned to π which quantifies the probability a SNP is associated with a phenotype. In this case the same value of π was used for all SNPs within a trait and thus π reflects the overall proportion of SNPs assumed to be associated with a particular phenotype. Here, π was calculated for each trait separately by dividing the total number of significant SNPs from the single SNP regression model for that trait in the sires by the total number of SNPs in the analysis (i.e. 40,668 SNPs). Additional BayesB analyses were run with different values for π to test the sensitivity of the associations to this prior; these priors included twice or half the original prior and are provided in Table 3. Further values of π (e.g. π = 0.0001, 0.00001) were tested in the Bayesian approach to gauge the influence of prior values on the Bayesian approach. The Markov Chain Monte Carlo chains were used to sample from the posterior distribution and these were run for 300,000 cycles with the first 150,000 cycles discarded as burn-in. Convergence was determined visually by plotting the model log-likelihood for all iterations. The number of times a SNP had a non-zero effect after the burn-in was recorded for each SNP; this number was then divided by the total number of iterations after burn-in to give the posterior probability of association (PPA). The PPA, ranging from zero (no association) to one (highly associated), indicated the strength of evidence from the posterior distribution that the particular SNP was associated with the phenotype in a manner roughly equivalent to a frequentist p-value.

**Table 3:** Values of π (prior) used in the Bayesian analysis in the sires

	S.S. in Sires	Total	SSR	SSR/2	SSRx2
Milk Yield	370	40,668	0.0091	0.0045	0.0182

Fat Yield	370	40,668	0.0091	0.0045	0.0182

Protein Yield	385	40,668	0.0095	0.0047	0.0189

Fat %	216	40,668	0.0053	0.0027	0.0106

Protein %	229	40,668	0.0056	0.0028	0.0113

SCS	9	40,668	0.0002	0.0001	0.0004

Fat % = fat percentage; Protein % = protein percentage; SCS = somatic cell score; S.S. in Sires = number of significant SNPs from the single SNP regression model in the sires for a particular trait; Total = total number of SNPs in the analysis; SSR = value of π based on the number of significant SNPs from the single SNP regression model in the sires (i.e. for milk yield, 370/40668 = 0.0091); SSR/2 = half the value of π than that of the SSR prior; SSRx2 = twice the value of π than that of the SSR prior

For the Bayesian analysis three different priors were used for each trait; these priors represent the proportion of SNPs believed to affect the particular trait. The SSR prior for a trait is calculated by dividing the total number of significant SNPs from the single SNP regression analysis in the sires by the total number of SNPs in the analysis (i.e. 40,668). The other two priors, SSR/2 and SSRx2 are simply the half and double the SSR prior respectively

### Assignment of SNP to genes

Genotypic data from the sires consisting of 40,668 SNPs typed across 914 individuals were used to estimate haplotype blocks. The 'Solid Spine of LD' method (based on the D prime statistic) in Haploview [60], with default settings, was used to estimate the blocks. Bovine gene start and end positions were sourced from Ensembl [61] based on build BTAU 4.0. SNPs were assigned to genes using an in-house Perl [62] script which worked in the following manner. If a SNP was located within a particular haplotype block then all genes that overlapped with that haplotype block were assigned to the SNP. Any SNP located outside a haplotype block was assigned to the closest gene. The assignment of SNPs to genes allowed a list of associated genes to be generated for each trait from the significant SNPs detected across all statistical approaches in the sires and cows.

### Checking for overlap with known QTL regions

All significant associations were examined to determine if they overlapped with known QTL regions using an in-house Perl script; if any significant SNP did not overlap with a known QTL region it was considered novel. The location of known QTL regions for the milk production traits and SCS were sourced from Cattle QTLdb [28]. We used the 'QTL Span' and/or 'QTL Peak Location' values in CattleQTLdb to define the boundaries of these known QTL. The keywords used for searches in the database were 'milk yield', 'milk fat yield', 'milk protein yield', 'milk fat percentage', 'milk protein percentage' and 'somatic cell score' for the traits milk yield, fat yield, protein yield, fat percentage, protein percentage and somatic cell score, respectively.

## Competing interests

The authors declare that they have no competing interests.

## Authors' contributions

DPB, DJL and AGF designed research; BKM performed the research, analyzed data and wrote the paper with DJL and DPB; JFK, DGB provided genotypic and phenotypic data; EKF carried out the DNA extraction. All authors read and approved the final manuscript.

## Supplementary Material

Additional file 1 **Summary statistics for the cow dataset**. This file contains summary statistics for the phenotypic data in the cow validation dataset mentioned in the manuscript. Summary statistics include the total number of phenotypic records (N), mean and standard deviation (σ) for each trait in the cows. Phenotypes in the cows are expressed as yield deviations on a PTA scale. Click here for file

Additional file 2 **Correlations between phenotypes for the cow dataset**. This file contains Pearson correlations between phenotypes in the cow dataset expressed as yield deviations on a PTA scale. Click here for file

Additional file 3 **All Significant Associations**. This file contains all significant SNPs detected across all the analyses in the sires and cows. Significant associations are presented separately for each analysis with separate worksheets for each trait. Click here for file

Additional file 4 **Cow Dataset Materials and Methods**. This file contains information on the materials and methods used for the cow validation dataset mentioned in the manuscript. Click here for file
