# Supplementary material for: Genome-wide associations for milk production and somatic cell score in Holstein-Friesian cattle in Ireland
Source: BMC Genet. 2012 Mar 26;13:21. doi: 10.1186/1471-2156-13-21 (PMC3361482; doi:10.1186/1471-2156-13-21)
Supplement: Additional file 4 — Cow Dataset Materials and Methods. This file contains information on the materials and methods used for the cow validation dataset mentioned in the manuscript. [file 1471-2156-13-21-S4.PDF]

# Cow Dataset Materials and Methods

## Cow DNA Extraction

Frozen blood samples were sourced from commercial Holstein-Friesian cows identified through the national database as having high reliability for milk production. DNA extraction and purification techniques were undertaken using the ABI Prism™ 6100 Nucleic Acid PrepStation (Applied Biosystems, Foster City, CA). The following protocol was used; 1) 15μL of proteinase K solution (20 mg/mL) was placed in 2-mL microcentrifuge tubes, 2) 85μL of PK digestion buffer was added, 3) 50μL of frozen whole bovine blood was added to the proteinase K and PK digestion buffer mixture and incubated at 58˚C for 20 minutes, 4) cell lysis was completed by adding 500μL of BloodPrep DNA purification solution. The DNA lysates were then passed across a 96-well DNA purification tray which was under vacuum. DNA was then washed to remove proteins and other contaminants using BloodPrep DNA wash solution. The purified DNA then underwent a two-step elution process at room temperature.

## Cow Genotypic and Phenotypic Data

In total, 563 Holstein-Friesian cows were genotyped for 54,001 biallelic SNPs using the Illumina BovineSNP50 Genotyping Beadchip (Illumina Inc., San Diego, CA). The 230 SNPs that were previously identified from a large dataset not to conform to Mendelian inheritance were also discarded. SNP positions were based on BTAU 4.0. The remaining SNPs were removed in the following order; if SNPs were on the X chromosome or whose position on the genome was unknown (n=2,419), if SNPs were monomorphic (n=4,874), if SNPs had a minor allele frequency ≤5% (n=4,907), if greater than 5% of SNP calls were missing (n=1,977) or if there was poor SNP clustering (n=451). Following edits 39,143 SNPs remained.

Yield deviations (YD) for milk yield, fat yield, protein yield, fat percentage, protein percentage and SCS along with their respective reliabilities were also available from the national genetic evaluation undertaken in January 2010 by the ICBF. The parental contribution to the reliability of each trait was removed. Only cows with an adjusted reliability (i.e. remove parental contribution to reliability) for milk production traits ≥65% or ≥40% for SCS were retained. In total 490 cows met these criteria for inclusion in the association analysis for milk production; 493 cows were included for SCS.
